# Quality of interhospital transport of critically ill patients: a prospective audit

**DOI:** 10.1186/cc3749

**Published:** 2005-07-01

**Authors:** Jack JM Ligtenberg, L Gert Arnold, Ymkje Stienstra, Tjip S van der Werf, John HJM Meertens, Jaap E Tulleken, Jan G Zijlstra

**Affiliations:** 1 Internist-intensivist, Intensive and Respiratory Care Unit (ICB), Department of Internal Medicine, University Medical Center Groningen, Groningen, The Netherlands; 2 Intensive Care Nurse, Intensive and Respiratory Care Unit (ICB), Department of Internal Medicine, University Medical Center Groningen, Groningen, The Netherlands; 3 Senior resident, Department of Internal Medicine, University Medical Center Groningen, Groningen, The Netherlands; 4 Pulmonologist-intensivist, Intensive and Respiratory Care Unit (ICB), Department of Internal Medicine, University Medical Center Groningen, Groningen, The Netherlands; 5 Anesthesiologist-intensivist, Intensive and Respiratory Care Unit (ICB), Department of Internal Medicine, University Medical Center Groningen, Groningen, The Netherlands

## Abstract

**Introduction:**

The aim of transferring a critically ill patient to the intensive care unit (ICU) of a tertiary referral centre is to improve prognosis. The transport itself must be as safe as possible and should not pose additional risks. We performed a prospective audit of the quality of interhospital transports to our university hospital-based medical ICU.

**Methods:**

Transfers were undertaken using standard ambulances. On departure and immediately after arrival, the following data were collected: blood pressure, heart rate, body temperature, oxygen saturation, arterial blood gas analysis, serum lactic acid, plasma haemoglobin concentration, blood glucose, mechanical ventilation settings, use of vasopressor/inotropic drugs, and presence of venous and arterial catheters. Ambulance personnel completed forms describing haemodynamic and ventilatory data during transport. Data were collected by our research nurse and analyzed.

**Results:**

A total of 100 consecutive transfers of ICU patients over a 14-month period were evaluated. Sixty-five per cent of patients were mechanically ventilated; 38% were on vasoactive drugs. Thirty-seven per cent exhibited an increased number of vital variables beyond predefined thresholds after transport compared with before transport; 34% had an equal number; and 29% had a lower number of vital variables beyond thresholds after transport. The distance of transport did not correlate with the condition on arrival. Six patients died within 24 hours after arrival; vital variables in these patients were not significantly different from those in patients who survived the first 24 hours. ICU mortality was 27%. Adverse events occurred in 34% of transfers; in 50% of these transports, pretransport recommendations given by the intensivist of our ICU were ignored. Approximately 30% of events may be attributed to technical problems.

**Conclusion:**

On aggregate, the quality of transport in our catchment area carried out using standard ambulances appeared to be satisfactory. However, examination of the data in greater detail revealed a number of preventable events. Further improvement must be achieved by better communication between referring and receiving hospitals, and by strict adherence to checklists and to published protocols. Patients transported between ICUs are still critically ill and should be treated as such.

## Introduction

Transfer of critically ill patients to the intensive care unit (ICU) of a tertiary referral centre is intended to improve prognosis. The transport itself must be as safe as possible and should not pose additional risks to the patient. Circulatory or ventilatory problems may arise in the ambulance as well as during transportation inside the hospital [1-3]. Monitoring capabilities are limited during transportation, and fewer (and less skilled) 'hands' are available on the road as compared with the ICU environment.

We accept 90–100 patient transfers each year from the ICUs of other hospitals. In our region patients are generally transported in standard ambulances; it is the responsibility of the referring hospital to ensure that a safe transfer takes place. We conducted a prospective audit of the quality of these transports, addressing the following questions. Did vital variables, documented before transfer, pass critical thresholds during transportation? Were changes in vital variables dependent on duration and/or length of transport? Did patients dying during or shortly after transfer have other vital variables before or during transport than did patients who survived the first 24 hours? Would it be possible to predict which patients will not benefit at all from transfer? Finally, what was the frequency of adverse incidents related to transfer?

We anticipated that answers to these questions would help us to decide whether an upgrade to transports in our area is needed, for example putting a mobile intensive care unit (ICU) from our university hospital into action.

## Materials and methods

A communication was send to the referring hospitals and ambulance services explaining the aims of the study. We clarified the protocol at different locations. Once these services and hospitals had agreed to participate, the study was started. In all cases, patients were transferred after telephone consultation with the supervising staff member of our ICU, who authorized the admission. The referring hospital was advised to stabilize the patient as much as possible and to send a skilled physician with the patient. Predefined study variables in 100 consecutive ICU transports were recorded just before departure and immediately after arrival. The following data were collected: blood pressure, heart rate, body temperature, oxygen saturation, arterial blood gases, serum lactic acid, plasma haemoglobin concentration, blood glucose, mechanical ventilation settings, use of vasopressor/inotropic medication, and presence of venous and arterial catheters. Ambulance personnel completed forms describing haemodynamic and ventilatory data during transport. Blood sampling and data acquisition on arrival were performed with the patient still on the ambulance stretcher (if this was considered safe), before changes to the 'on the road' therapy were instituted. Immediately thereafter, the patient was moved to the ICU bed and connected to the ICU ventilator. Data were instantly noted on a simple data sheet, and checked and collected by our research nurse.

The local medical ethics committee was informed and approved the design of our study.

### Statistical analysis

We tested, for each parameter, whether the value beyond a predefined threshold on departure differed from the value beyond the threshold on arrival, using the the McNemar test. This test is typically used in a repeated measures situation, in which each subject's response is elicited twice, once before and once after a specified event (in this case transfer) occurs. For calculations of the change in number of vital variables beyond the threshold occurring as a result of transport, variables were included – when available – both from before and after transport. For each individual the total number of vital variables beyond threshold before and after transport was determined. Whether there was a difference between these two time points was tested using the Wilcoxon signed ranks test. *P *< 0.05 was considered statistically significant. Data were analyzed using SPSS for Windows version 12.0 (SPSS Inc, Chicago, IL, USA).

## Results

In total, 100 consecutive transfers of ICU patients were evaluated over a 14-month period.

### Transport characteristics

Patient transport characteristics are summarized in Table 1. Most transfers (96) were from 18 regional hospitals in the north eastern part of The Netherlands; four were from four ICUs located elsewhere in The Netherlands. Three ICUs transferred 10 or more patients; five ICUs transferred five to nine patients; and the others transferred between one and four patients. More than one-third arrived during the night shift (i.e. between 17.00 and 08.00 hours). An ICU nurse was present in 23% of transports and a physician in 57%. Blood was drawn within 6.4 ± 9 min (mean ± standard deviation) after arrival at our ICU.

### Diagnoses

Respiratory problems (e.g. oxygenation problems during mechanical ventilation or weaning difficulties) were the most common reason for transfer (Table 2). Severe multiple organ failure and sepsis together were responsible for 35% of transfers, in part because of a need for renal replacement therapy. Four patients with gastrointestinal tract bleeding were transferred because trained interventional endoscopists were not available in the admission hospital. Shortage of ICU capacity was cited as the reason for transport on only a few occasions. A diagnostic problem existed in more than 30% of cases.

### Patient characteristics on arrival

The characteristics of patients on arrival are summarized in Table 3. Sixty-five per cent of patients were mechanically ventilated and 38% were on vasoactive drugs.

### Vital parameters

Variables on departure and arrival are summarized in Table 4. The percentage of patients arriving with values beyond predefined critical thresholds is shown. We defined these thresholds as clinically relevant deteriorations, based on thresholds cited in the literature (e.g. the haemoglobin threshold cited in the study by Hebert and coworkers [4] and in the TRICC (Transfusion requirements in critical care) trial [5] or thresholds used in clinical practice in The Netherlands. The number of patients in whom a critical threshold was reached during transport was calculated (with normal values on departure but values beyond critical thresholds at arrival indicating a worsening in the patient's condition during transfer).

The median number of variables beyond threshold was 2 (of the 12 mentioned in Table 4), both before and after transfer. The maximum number of variables beyond threshold in one patient was 6 (after transfer). Thirty-seven per cent of patients exhibited an increased number of variables beyond threshold after transport as compared with before transport (23% had one parameter more after transport, 9% had two more, 3% had three more, 1% had four more and 1% had five more); 34% had an equal number beyond threshold before and after transport; and 29% had a lower number beyond threshold after transport. These differences were not statistically significant (*P *= 0.182, by Wilcoxon signed rank test).

Patients in whom there was a greater number of variables beyond threshold after transport than before transport did not have a longer transportation time than did the other patients (median transport time: 40 min versus 38 min, respectively; *P *= 0.76, by Mann–Whitney U-test). Six patients (6%) died within 24 hours after arrival. These patients had a median of 2.5 variables beyond threshold at departure, and a median of 3 vital parameters beyond threshold at arrival. For the patients who did not die within 24 hours there were 2 variables beyond threshold both before and after transport (*P *= 0.74, by Mann–Whitney U-test). ICU mortality was 27%.

### Adverse events

Events were recorded in 34 out of 100 transfers (examples of such events are sumarized in Table 5). The impact of various events was graded as follows: grade 1 = deviation from ambulance guidelines/local protocols/advice from tertiary centre; grade 2 = of vital importance – immediate action needed on arrival; and grade 3 = of vital importance – immediate action needed on arrival – event probably avoidable.

In summary, adverse events occurred in 34% of transfers. In 50% of these transports recommendations for safe transport of the patient, given by the intensivist of our ICU, were ignored. We estimate that 70% of events could have been avoided by better preparation for the transfer. Approximately 30% of events could be attributed to technical problems during transport; some of these could have been prevented (e.g. shortage of oxygen on the road).

## Discussion

In this prospective study, changes in parameters for major worsening during transport never achieved statistical significance (Table 4). Based on this, one could conclude that the quality of transport in our catchment area, carried out with standard ambulances, is sufficient. However, evaluation of data for individual patients showed some serious deteriorations during transport; in 34% of transports events occurred, some of which had vital complications. Of course, this could be because the patients were critically ill. Another cause of deterioration could be the occurrence of adverse events during transport. In 50% of the transports with events, recommendations for transport of the patient – given by the intensivist of our ICU – were ignored. We estimate that 70% of events could have been avoided by better preparation and communication before transfer. This may represent poor clinical care by the referring centres, perhaps caused by underestimation of the risks associated with ICU tranfers or overestimation of the skills of ambulance personnel. Another reason may be that we had no standard protocol for giving feedback to the referring physician after the transfer had been performed. Thirty per cent of events could be attributed to technical problems during transport.

ICU mortality in our study group was 27%; because 73% survives one could state, that our way of selecting patients for referral is adequate and that these 73% benefit from admission to our ICU. However, no data are available on mortality in comparable patients in our region, who were *not *transported. The APACHE II score on arrival did not differ from our average ICU population; however, the APACHE score of the study group may be affected by prior stabilisation during admission in the referring hospital. Mortality of our total ICU population is 18%; this illustrates the fact that APACHE score may not be an adequate instrument to predict mortality in transferred patients from other hospitals [6]. Durairaj et al -in a large study in > 3000 transferred ICU patients – used diagnostic category and comorbidity scores, which showed a better correlation with morbidity and mortality in transferred patients [7].

We are concerned about the observed lack of preparation before transfer of patients. Although we consistently advised that a skilled physician accompany the patient, a number of patients arrived without a doctor. Simply adherence to existing ambulance checklists would have avoided a few events, for example equipment failures, incomplete supplies, shortage of oxygen or batteries, and drug administration errors.

We do not know whether a special retrieval team using a mobile ICU would improve the quality of transports in our catchment area. Several positive experiences with special retrieval teams have been reported [8,9]. In a study conducted by Bellingan and coworkers [7] transports by a specialist retrieval team, compared with standard ambulance transport with a doctor from the referring hospital, resulted in more stabile transports and a reduction in mortality during the first 12 hours from 7.7% to 3%. ICU mortality was not significantly different (35% versus 28%). It seems logical to use a specialist team and a mobile ICU for transport of more severely ill patients [10], but we were unable to find reports of pretransport parameters that could predict which patients will deteriorate during transfer and who may benefit from a retrieval ream with a mobile ICU.

Guidelines for safe ICU transfers have been reported by The Netherlands Society of Intensive Care, among others [11-13], and there are new regional ambulance guidelines that require a skilled physician to accompany each ventilated patient; if this is not possible the patient will not be transported. Based on our own findings and the new guidelines, our recommendations to the referring centre for the transfer of patients have become more strict.

## Conclusion

Before deciding to transport a critically ill patient, it must be borne in mind that such a transfer has its own risks. Such risks will become more prominent in the near future because of the tendency to centralize advanced health support to a few regional centres. Further improvement must be achieved by better communication between the referring and receiving hospital before transport is initiated, and by strict adherence to checklists and to published guidelines. Patients transported between ICUs are still critically ill patients and should be treated as such. Whether these measures will render the use of a mobile ICU in our area unnecessary is not yet known.

## Key messages

• 	Interhospital transfer of critically ill patients must be as safe as possible and should not pose additional risks.

• 	Seventy per cent of events that occurred could have been avoided by better preparation for the transfer.

• 	Further improvement may be achieved by better communication between the referring and receiving hospital before the transport is initiated, and by strict adherence to checklists and published guidelines.

## Abbreviations

APACHE = Acute Physiology and Chronic Health Evaluation; ICU = intensive care unit.

## Competing interests

The author(s) declare that they have no competing interests.

## Figures and Tables

**Table 1:** Transport characteristics

Characteristic	Value
Distance (km)	57 ± 43
Transporting time (min)	47 ± 30
Arrival 17.00–08.00 hours (%)	37
Ambulance nurse (%)	100
+ ICU nurse (%)	23
+ Physician (and ICU nurse) (%)	57
Blood sampling after arrival (min)	6.4 ± 9

Data are given as mean ± standard deviation or as percentage.

**Table 2:** Transfer diagnosis

Reason for transfer	%
Respiratory problems	32
Multiple organ failure	25
Sepsis	10
Cardiac	8
(Neuro)surgical problems	8
Gastrointestinal bleeding	4
Intoxication	4
Other diagnosis	9

'Other diagnoses' include end-stage liver failure (*n *= 1), HELLP (haemolysis–elevated liver enzymes–low platelets) syndrome (*n *= 2), microangiopathic thrombotic syndrome (thrombotic thrombocytopenic purpura/haemolytic uremic syndrome; *n *= 2), Wegener's granulomatosis (*n *= 2), and pulmonary embolism (*n *= 2).

**Table 3:** Characteristics of patients on arrival

Characteristic	Value
Age (years)	54.7 ± 1.7
Sex (female/male)	49/51
Mechanically ventilated	65
Oxygen	
Mask	14
Nasal	21
Central venous line	47
Intra-arterial catheter	72
Peripheral venous line	96
Vasopressor/inotropic drugs	38
APACHE II score	12.6 ± 0.7

Data are expressed as a percentage (%) or as mean ± standard deviation. APACHE, Acute Physiology and Chronic Health Evaluation.

**Table 4:** Variables on departure and arrival

Variable	Departure (mean ± SD)	Arrival (mean ± SD)	Arrival (min–max)	Critical threshold	Beyond threshold on departure (%)	Beyond threshold during transport (%)	Beyond threshold on arrival (%)	*P*
Arterial pH	7.35 ± 0.17	7.36 ± 0.13	6.98–7.57	<7.20	7	-	13	0.18
Oxygen saturation (%)	94.2 ± 7.0	93.9 ± 7.3	68–100	<90	13	20	16	0.58
PCO_2 _(kPa)	13.5 ± 9.9	18.3 ± 14.7	4.30–71.8	<8	12	-	16	0.45
PCO_2 _(kPa)	5.6 ± 1.8	5.9 ± 2.3	2.80–12.91	>6.0	35	-	35	1.00
SBP (mmHg)	120.8 ± 22.8	126.9 ± 30	60–210	<90, >180	7, 0	14, 3	11, 3	0.09
DBP (mmHg)	64.4 ± 15.9	68.43 ± 18.4	37–145	<50, >110	14, 1	14, 3	14, 2	1.00
Heart rate (beats/min)	103.5 ± 23.8	103.9 ± 23.3	50–160	<50, >120	0, 30	2, 31	0, 28	0.82
Temperature (°C)	37.7 ± 1.3	37.04 ± 1.3	32.0–40.4	<36.0	8	-	12	0.29
Lactate (mmol/l)	1.9 ± 2.5	2.1 ± 1.9	0.6–13.2	>3.0	10	-	8	1.00
Haemoglobin (mmol/l; g/dl × 1.6)	6.9 ± 1.5	6.6 ± 1.4	3.4–10.0	<5.0	6	-	11	0.06
Glucose (mmol/l)	9.0 ± 5.0	7.9 ± 4.3	3.4–25.5	<4.0, >12.0	15	-	15	1.00
HCO_3_^-^(mmol/l)	23.2 ± 6.3	23.9 ± 7.0	5.1–44.0	<20	36	-	28	0.09

*P values were calculated using the McNemar test. -, not measured during transport; DBP, diastolic blood pressure; PCO_2_, partial carbon dioxide tension; PO_2_, partial oxygen tension; SBP, systolic blood pressure.

**Table 5:** Examples of recorded adverse events

Transfer characteristics	Adverse event	Severity (grade 1–3)^a^
Pulmonary embolus	PaO_2 _on departure 4.2 kPa; not intubated; PaO_2 _on arrival 4.7 kPa	3
Oesophageal bleeding	Only one peripheral intravenous line; no accompanying physician; active bleeding; PaO_2 _on arrival 6.7 kPa	3
Sepsis, rhabdomyolysis	RI; shock on arrival	2
Imminent RI; Wegener's granulomatosis	No blood pressure measured on the road (160 km); PaO_2 _on arrival 6.7 kPa, SaO_2 _86%	1
Pulmonary embolus	No accompanying physician; RI on arrival	2
ARDS, MOF	SaO_2 _93% at departure, 69% on arrival	3
Streptococcal pneumonia/sepsis; imminent RI	Not intubated (despite advice); norepinephrine via peripheral intravenous line	3
Sleep apnoea syndrome; RI	PaO_2 _on departure 6.9 kPa; during transport SaO_2_↓ 74% and cardiac ischaemia; no physician	3
Haemorragic shock; mechanical ventilation	No accompanying physician; active bleeding (3 units packed cells on the road); oxygenation problems	3
Infectious endocarditis; mechanical ventilation	No physician; haemodynamically unstable on the road	1
Septic shock; imminent RI	Not intubated (despite advice); RI on arrival	3
Septic shock; MOF	Norepinephrine via peripheral intravenous line	2
Suicide attempt (benzodiazepine)	Deep coma; not intubated; apnoea *en route*; cyanotic on arrival	3
Postsurgical; mechanical ventilation	Oxygen supply breakdown before arrival	3
COPD, pneumonia	Shortage of oxygen before arrival	3
Haemodyalisis postsurgical	No blood pressure measured on the road	1
Active bleeding digestive tract	Only one peripheral intravenous line	1
ARDS; mechanical ventilation	Ambulance breakdown; 40 min delay	1

^a^Grades of severity: grade 1 = deviation from guidelines/protocol; grade 2 = of vital importance – immediate action needed on arrival; and grade 3 = of vital importance – immediate action needed on arrival – event probably avoidable. ARDS, acute respiratory distress syndrome; MOF, multiple organ failure; PaO_2_, arterial oxygen tension; RI, respiratory insufficiency (imminent need for mechanical ventilation); SaO_2_, arterial oxygen saturation.
